# *In Silico* Identification of Natural Product-Based Inhibitors Targeting IL-1β/IL-1R Protein–Protein Interface

**DOI:** 10.3390/molecules28134885

**Published:** 2023-06-21

**Authors:** Ting-ting Liu, Yan-kun Chen, Muhammad Adil, Mazen Almehmadi, Fahad M. Alshabrmi, Mamdouh Allahyani, Ahad Amer Alsaiari, Pei Liu, Muhammad Raheel Khan, Qinghua Peng

**Affiliations:** 1 School of Chinese Medicine, Hunan University of Chinese Medicine, Changsha 410200, China; 2 Department of Biological Sciences, National University of Medical Sciences (NUMS), Rawalpindi 46000, Pakistan; 3 Institute of Biology, Biotechnology and Environmental Protection, Faculty of Natural Sciences, University of Silesia in Katowice, 40-007 Katowice, Poland; 4 Department of Clinical Laboratory Sciences, College of Applied Medical Sciences, Taif University, Taif 21944, Saudi Arabia; 5 Department of Medical Laboratories, College of Applied Medical Sciences, Qassim University, Buraydah 51452, Saudi Arabia; 6 Centre of Polymer and Carbon Materials, Polish Academy of Sciences, ul. Sklodowskiej-Curie 34, 41-819 Zabrze, Poland; 7 Department of Chemical Sciences, Joint Doctoral School, Silesian University of Technology, 44-100 Gliwice, Poland

**Keywords:** IL-1β, virtual screening, molecular docking, MD simulation, natural products

## Abstract

IL-1β mediates inflammation and regulates immune responses, cell proliferation, and differentiation. Dysregulation of IL-1β is linked to inflammatory and autoimmune diseases. Elevated IL-1β levels are found in patients with severe COVID-19, indicating its excessive production may worsen the disease. Also, dry eye disease patients show high IL-1β levels in tears and conjunctival epithelium. Therefore, IL-1β signaling is a potential therapeutic targeting for COVID-19 and aforementioned diseases. No small-molecule IL-1β inhibitor is clinically approved despite efforts. Developing such inhibitors is highly desirable. Herein, a docking-based strategy was used to screen the TCM (Traditional Chinese Medicine) database to identify possible IL-1β inhibitors with desirable pharmacological characteristics by targeting the IL-1β/IL-1R interface. Primarily, the docking-based screening was performed by selecting the crucial residues of IL-1β interface to retrieve the potential compounds. Afterwards, the compounds were shortlisted on the basis of binding scores and significant interactions with the crucial residues of IL-1β. Further, to gain insights into the dynamic behavior of the protein–ligand interactions, MD simulations were performed. The analysis suggests that four selected compounds were stabilized in an IL-1β pocket, possibly blocking the formation of an IL-1β/IL-1R complex. This indicates their potential to interfere with the immune response, making them potential therapeutic agents to investigate further.

## 1. Introduction

The term inflammation has long been known to share epidemiological and molecular grounds with the theory of disease [1]. The understanding of the intimate relationship underlying inflammatory processes and disease came into existence in 2004 when the ras gene involved in cancer was found to have a significant role in chronic inflammation [2]. By then, a spate of research papers had come along highlighting the role of inflammatory pathways in the progression and regulation of diseases such as cancer [3]. One such inflammatory pathway involves the interleukin-1 family (IL-1F) of cytokines, which have been known to participate in the course of innate immunity and which are primarily manifested by inflammation [4,5,6]. Genetic studies have revealed the involvement of two related but distinct genes for the interleukin-1 family (i.e., IL1A and IL1B), both of which are located on the longer arm of chromosome 2, encoding for IL1-α and IL1-β, respectively [6,7]. The α and β forms of the cytokine get transcribed and translated into 271 and 269 amino acid precursors (small mature proteins), respectively, which tend to exert their effects via the same cell surface receptor (i.e., IL-1 receptor type-1), triggering a cascade of inflammatory mediators, chemokines, and other cytokines [8,9].

The current research primarily focuses on IL-1β, which is regarded as a pro-inflammatory cytokine and has been reported to modulate severe and sustained inflammation. Unlike IL1-α, which is constitutively expressed among healthy individuals, IL-1β requires a series of events prior to the initiation of the inflammation cascade [10]. This involves the stimuli received in the shape of microbial epitopes/products; however, other cytokines, including IL-1α, IL-18, and tumor necrosis factor (TNF), as well as IL-1β itself, can induce IL-1β transcription [11]. Despite the fact that IL-1α is active in its precursor form, IL-1β needs to be processed, involving the action caspase-1 protein (present in procaspase-1 form and activated by complex formation with “NOD-like” receptor-3 (NLRP3) in the shape of an inflammasome), which cleaves the amino terminal of the molecule, releasing active IL-1β [12,13,14,15,16,17].

Due to the significant role played by IL-1β in triggering the inflammation cascade, it can be considered a potential emerging target for the treatment of chronic diseases [6]. IL-1β plays a crucial role in the pathogenesis of dry eye disease. Numerous investigations have demonstrated that the concentration of IL-1β is increased in both the tears and conjunctival tissues of individuals affected by dry eye disease, strongly suggesting the involvement of this inflammatory mediator in the disease’s development [18,19]. IL-1β’s impact on dry eye disease is complex and multifactorial, as it can affect various aspects of the disease’s pathophysiology. For instance, IL-1β can trigger the death of corneal epithelial cells, hinder the production of mucins and other tear components, promote the expression of matrix metalloproteinases (MMPs), and damage the ocular surface barrier’s integrity [20]. Furthermore, IL-1β can activate sensory nerve fibers located in the cornea and conjunctiva, resulting in the perception of pain and discomfort that characterizes dry eye disease [21]. Given its crucial role in the disease’s pathogenesis, targeting IL-1β and its downstream inflammatory pathways represents a promising therapeutic strategy for dry eye disease. Numerous studies have discussed the potential of IL-1β inhibitors in mitigating dry eye disease’s signs and symptoms [22].

On a similar note, IL-1β inhibitors have also been reported to play a substantial role in the prevention of COVID-19. In the case of SARS-CoV-2 infection, the epithelial damage to the cells causes the outburst of IL-1β, which tends to recruit inflammatory cells, predominantly monocytes, which further induces the secretion of IL-1β cytokine, triggering the autoinflammation loop [23,24]. To date, a number of IL-1β blockers have been investigated by the FDA for the treatment of COVID-19 [25,26]. Keeping aside the role of IL-1β protein as a potential therapeutic target for dry eye disease and COVID-19, its inhibitors have also been implicated to have a central role in the treatment of myeloma, rheumatoid arthritis, amyotrophic lateral sclerosis (ALS), etc. [27,28,29,30].

Hence, if chronic inflammation does underlie these many diseases, it would make sense to recognize it as a condition that should be treated in its own right as a preventative therapy. In that regard, the present research primarily focuses on the identification and screening of potential drug candidates against IL-1β in complex with the type-1 IL-1 receptor (IL-1R). The research predominantly takes advantage of computational biology by exploring its avenues to present a concrete framework to obtain lead compounds that can be further validated through *in vitro* studies.

## 2. Results

### 2.1. Docking-Based Virtual Screening

**Figure 1:**
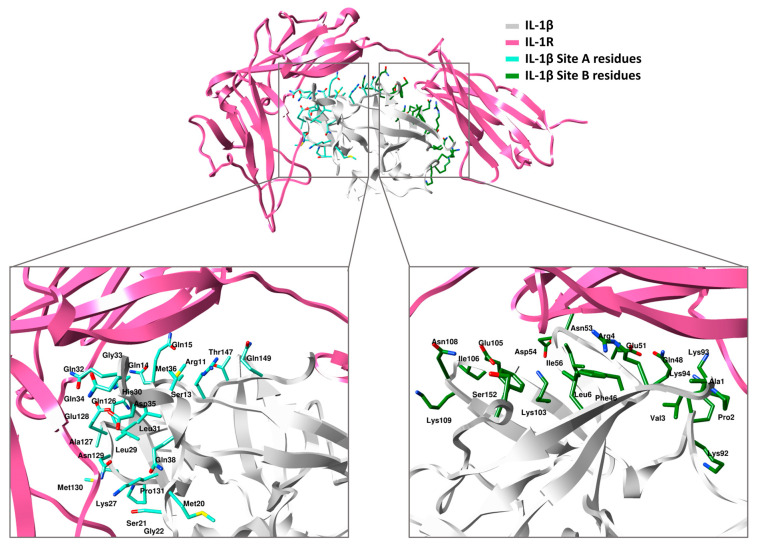
IL-1β in complex with IL-1R. The amino acid residues of IL-β site A and site B are indicated and labeled accordingly.

According to previous research, IL-1β contains two active sites, known as site A and site B. Site A interacts with the β-strand, a1, a2, b2, and the b2-c2 loop located in domains 1 and 2 of the receptor. This interaction involves specific amino acid residues, including Arg11, Ser13-Gln15, Met20-Gly22, Lys27, Leu29-Met36, Gln38, Gln126-Pro131, Thr147, and Gln149 (Figure 1) [31]. Located at the top of the β-barrel structure, the second binding site on IL-1β is referred to as Site B and is composed of specific amino acid residues, including Ala1-Arg4, Leu6, Phe46, Gln48, Glu51, Asn53, Asp54, Ile56, Lys92-Lys94, Lys103, Glu105, Ile106, Asn108, Lys109, Phe150, and Ser152. Unlike Site A, Site B only interacts with domain 3 of IL-1R (Figure 1) [31]. The hydrophobic interactions that primarily drive the assembly of the IL-1β/IL-1RI complex pose a challenging obstacle for small-molecule replication and experimental methods. Therefore, in this study, computer-aided drug design methods were utilized to identify potential small molecules with anti-inflammatory potential that can target the protein–protein interactions involved in the formation of the IL-1β/IL-1RI complex. In this connection, a multistep screening campaign based on molecular docking was executed to extract the potential IL-1β binders from the traditional Chinese medicine (TCM) database.

Using the interfacial residues of site A as a docking grid, molecular docking was carried out for all the compounds (~36,000) prepared from the TCM database. The docking simulation results were then analyzed based on the capability of the docked compounds to inhibit the protein–protein interaction of IL-1β/IL-1RI in the best-ranking binding pose. In the first step of the screening, the docked compounds were subjected to the protein-ligand interaction fingerprint (PLIF) module of MOE to fingerprint the interactions with the crucial residues. PLIF is a method for summarizing the interactions between ligands and proteins using a fingerprint scheme. Hydrogen bonds, ionic interactions, and surface contacts are classified according to the residue of origin, and built into a fingerprint scheme that is representative of a given database of protein–ligand complexes. Upon generating fingerprints for the ligands in the TCM database, it was observed that the compounds interacted with several key residues, including Met20, Ser21, Gly23, Tyr24, Glu25, Lys27, His30, Gln32, Gln34, Asp35, Gln38, Leu67, Glu128, Asn129, Thr147, and Gln149. In the first step, ~5000 compounds were shortlisted for further analysis. In the second step of the screening, the compounds were shortlisted on the basis of binding score. Notably, the highest binding score obtained was −14.9 kcal/mol. Consequently, the compounds with a binding score within the limits of −14.9 to −6.0 kcal/mol were preferred, leading to ~700 simulated hits. It is worth mentioning to the readers that the cutoff for the binding score was chosen by referring to a previous study, which reported the docking scores of biologically active IL-1β inhibitors to be below −6.0 kcal/mol [32]. In the last step of the screening, the compounds were visually analyzed. This analysis involved examining the hydrogen bonding and hydrophobic interactions with the essential residues as well as assessing the complementarity between the ligand and the binding site of IL-1β. After careful inspection, the four compounds were found to be the most potential binders of IL-1β and designated as compound **1**, compound **2**, compound **3**, and compound **4**. The chemical structures of the selected compounds are depicted in Table 1.

### 2.2. Analysis of Binding Mode

**Figure 2:**
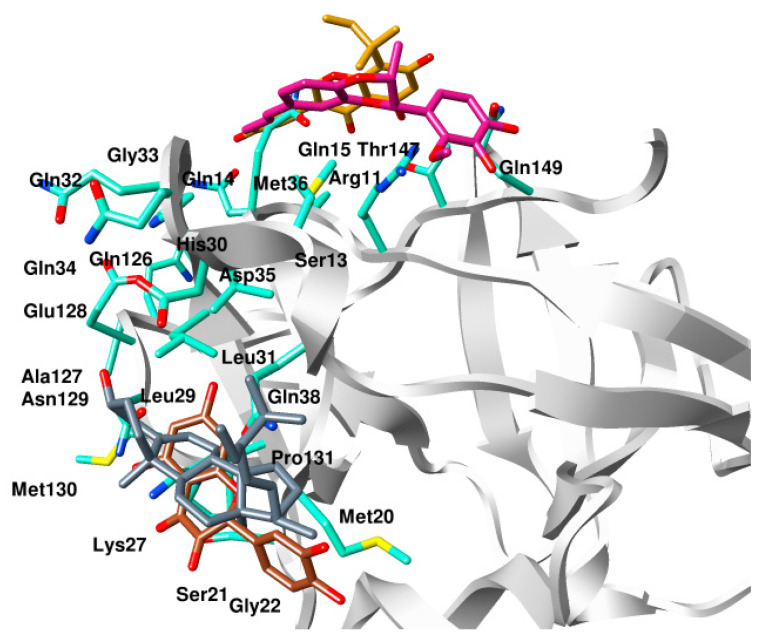
Superimposed binding modes of compound **1** (violet red), compound **2** (golden), compound **3** (brown), and compound **4** (slate blue) in the binding site A of IL-1β.

**Figure 3:**
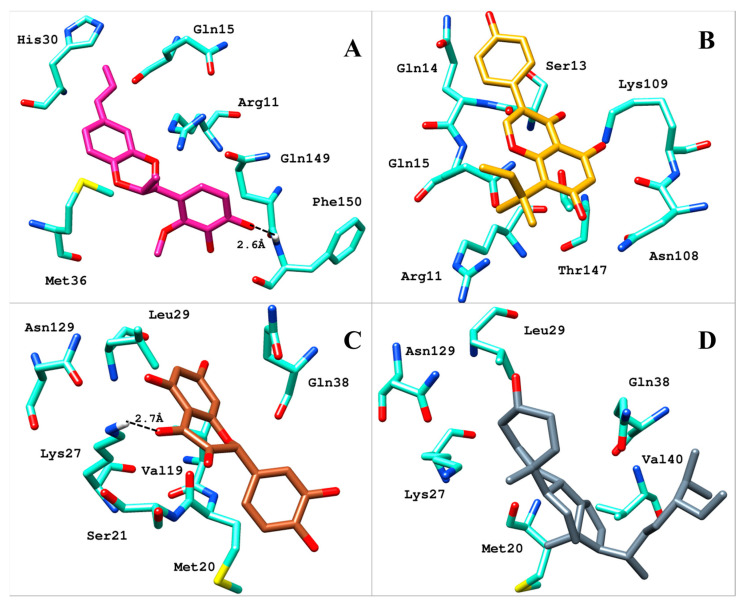
Binding mode of (**A**) compound **1**, (**B**) compound **2**, (**C**) compound **3**, and (**D**) compound **4** in the binding site of IL-1β. The residues of IL-1β surrounded by ligands are shown in aquamarine sticks while compounds are shown in different color sticks.

All four compounds bound to IL-1β with a significant binding score in the range of −14.19 kcal/mol to −10.10 kcal/mol (Table 1). The superimposed binding modes of all the compounds are shown in Figure 2. In the binding site of IL-1β, compound **1** resided well by mediating a number of significant interactions (Figure 3A). The hydroxyl group substituted on the phenyl ring established two hydrogen bond contacts with the peptide carbonyl of Phe150. Similarly, dioxane oxygen mediated polar interactions with the side chains of Arh11 and Met36. Likewise, the allylic chain of the compound was observed to mediate pi-interactions with the side chains of Gln15 and His30, while Gln149 was involved in the pi-interaction with the phenyl ring. Similarly, the docked position of compound **2**, in the binding site of IL-1β, demonstrated a network of polar interactions (Figure 3B). Similarly, the substituted hydroxyl groups on phenyl rings mediated polar interactions with Arg11, Ser13, Gln14, Asn108, Lys109, and Thr147. Similarly, the oxygen in the pyran ring of the compounds mediated a polar interaction with Gln15. In the case of compound **3**, the hydroxyl group substituted on the phenyl ring formed a hydrogen bond with the side chain of Lys27 (Figure 3C). Similarly, the hydroxyl groups and carbonyl group substituted on the pyran ring were also involved in mediating polar interactions with Ser21, Gln38, and Asn149. Phenyl rings were involved in mediating the pi-interactions with the side chains of Met20, Val19, and Leu29, whereas, compound **4** stabilized itself in the binding site of IL-1β by establishing significant interactions with the crucial residues (Figure 3D). Met20, Lys29, Leu27, Val40, Gln38, and Asn129 were observed to accommodate the compound by mediating a hydrophobic interaction.

### 2.3. Molecular Dynamics Simulation

The molecular docking studies demonstrated that the hit compounds interacted significantly with the crucial residues of site A and exhibited complementarity. However, molecular docking studies alone do not provide information on the time-dependent stability of interactions, the impact of solvent, and the intrinsic dynamics of the protein. As a result, molecular dynamics (MD) simulations were performed on four selected compounds in complex with IL-1β to investigate the stability of protein–ligand interactions. The analysis was carried out by examining parameters such as root mean square deviation (RMSD), root mean square fluctuation (RMSF), and radius of gyration (Rg).

**Figure 4:**
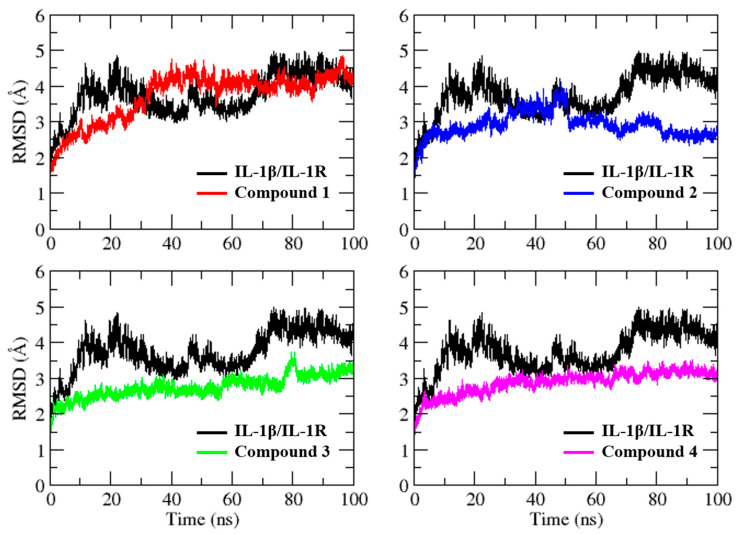
Root mean square deviation plot of IL-1β/IL-1R complex and selected virtual hits in complex with IL-1β during a 100 ns simulation. The RMSD values were calculated with respect to the initial structure of the complex, and the plot shows the time evolution of the deviation of the complex from its starting conformation.

The MD simulation of the IL-1β/IL-1R complex revealed a dramatic change from the initial structure, with ~5 Å of difference in RMSD (Figure 4). Interestingly, despite the small size of the selected hit compounds relative to the IL-1R, the binding of the ligand to the IL-1β binding site resulted in a more stable protein conformation and exhibited less variation throughout the simulation, with the exception of compound **1**, which could be attributed to its relatively lower docking score compared to that of the other compounds. For compound **1**, the RMSD showed a gradual increase of up to 4.5 Å during the first 40 ns of simulation, followed by another increase in the RMSD value during the final 10 ns. Similarly, for compound **2**, the RMSD increased gradually up to 4.0 Å during the first 50 ns of simulation. After the initial 50 ns, the system gradually achieved stability and remained stable until the end of the simulation, with minor fluctuations. For compound **3**, the system experienced fluctuations between 2 Å and 3 Å during the simulation, with a consistently stable RMSD except for frames between 75 and 85 ns, where some sharp fluctuation peaks were observed but still remained within an acceptable range. On the other hand, the complex of compound **4** showed the most stable RMSD below 3.5 Å with minimal variation, indicating that the system had converged well.

**Figure 5:**
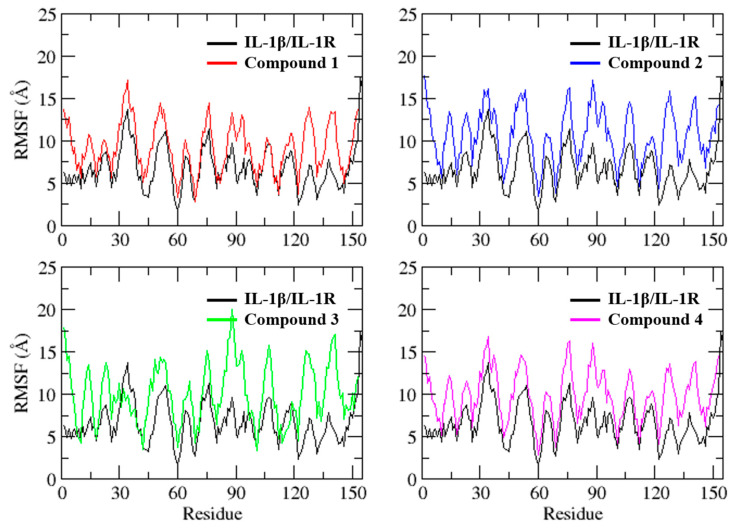
Root mean square fluctuations plot of IL-1β/IL-1R complex and selected virtual hits in complex with IL-1β during a 100 ns MD simulation. The plot shows the residue-wise RMSF values of the protein and ligand atoms, with the *x*-axis representing the residue index and the *y*-axis representing the RMSF values in angstroms.

To assess the impact of the ligand on the flexibility of IL-1β residues, RMSF was computed (Figure 5). Despite exhibiting comparable fluctuation patterns, the magnitude of the peaks varied among the different systems. Interestingly, the IL-1β/IL-1R complex residues displayed lower fluctuations than the chosen virtual hit complexes, indicating that it had relatively lower structural mobility. The average RMSF values for IL-1β/IL-1R, compound **1**, compound **2**, compound **3**, and compound **4** complexes were 6.4 ± 2.1 Å, 9.1 ± 2.8 Å, 10.6 ± 3.1 Å, 10.0 ± 3.4 Å, and 9.9 ± 2.9 Å, respectively. The differences in RMSF values between the IL-1β/IL-1R complex and the virtual hit complexes suggest that the ligands might affect the flexibility of the receptor residues differently. The higher RMSF values observed for the virtual hit complexes might be indicative of the ligand-induced structural perturbation of the receptor.

**Figure 6:**
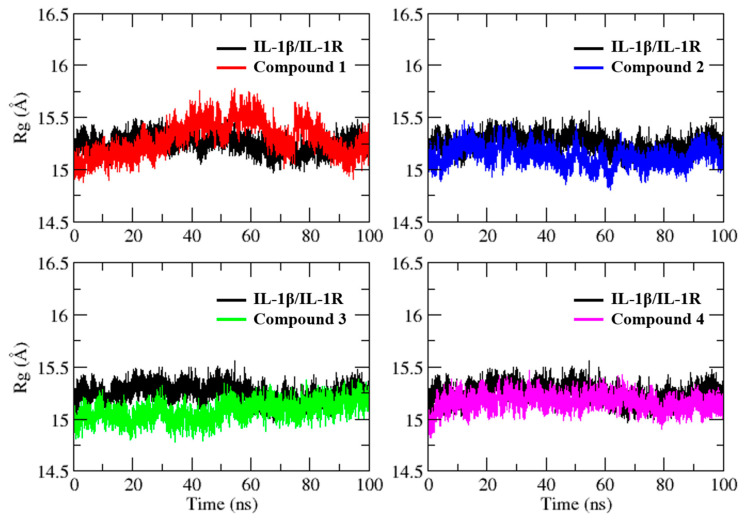
Radius of gyration plot for IL-1β/IL-1R complex and selected virtual hits in complex with IL-1β obtained from a 100 ns MD simulation, depicting the time evolution of the root mean square distance of the complex from its center of mass.

The assessment of the compactness of IL-1β in complex with its receptor and the selected hit compounds was conducted through the calculation of the radius of gyration (Rg), as shown in Figure 6. Interestingly, the Rg plots of all simulated systems exhibited stable values throughout the MD simulations, indicating that the overall compactness and structural integrity of the protein–ligand complexes were maintained. The average Rg scores for the IL-1β/IL-1R complex, compound **1**, compound **2**, compound **3**, and compound **4** were determined to be 15.2 ± 0.07 Å, 15.2 ± 0.15 Å, 15.1 ± 0.09 Å, 15.0 ± 0.08 Å, and 15.1 ± 0.07 Å, respectively. These results demonstrate that the binding of the hit compounds to the IL-1β binding site did not cause significant perturbation to the protein’s overall compactness.

## 3. Discussion

Natural products have been an important source of drugs for centuries. Many of the drugs used today are derived from natural products or were developed based on the structure of natural products [33]. In recent years, there has been growing interest in using TCM databases to identify new natural product leads for drug discovery [34]. Traditional Chinese medicine (TCM) is a holistic system of medicine that has been practiced for thousands of years in China and other parts of Asia. It involves the use of natural remedies, such as herbs, minerals, and animal products, as well as acupuncture and other techniques, to promote health and treat illness. TCM remedies are often used in combination to achieve a synergistic effect, and they are tailored to the individual patient based on their unique symptoms and constitution [35]. Research has been conducted to explore its potential for countering the inflammatory effects caused by IL-1β. For instance, baicalin, a flavonoid that is found in the roots of Scutellaria baicalensis Georgi, has been shown to inhibit the expression of IL-1β in several studies [36]. Similarly, the polyphenols curcumin and resveratrol, found in the spice turmeric and grapes, respectively, have been shown to have anti-inflammatory effects, and studies have found that they can inhibit the production of IL-1β [37,38]. Similarly, triptolide, a diterpene triepoxide found in the herb Tripterygium wilfordii Hook F., has also shown the potential to inhibit the production of IL-1β in several studies [39]. In a previous study, Stierle et al. reported the biological activity of certain isolated berkeleyones against IL-1β, demonstrating low micromolar activity [40]. Therefore, in the present study, we aimed to identify small molecules from the TCM database [41] that can target the protein–protein interactions involved in the formation of the IL-1β/IL-1R complex using docking-based virtual screening and MD simulation. Initially, we employed molecular docking to screen approximately 36,000 compounds from the TCM database. The docking simulation results were analyzed based on the ability of the docked compounds to inhibit the protein–protein interaction of IL-1β/IL-1RI in the best-ranking binding pose. As a result of this analysis, we identified four compounds (compound **1**, compound **2**, compound **3**, and compound **4**) that exhibited significant binding scores and displayed high potential as IL-1β binders. The selection of these compounds was based on their docked positions’ ability to impede protein–protein interactions, a binding score within the range of −14.9 to −6.0 kcal/mol, and in-depth visual inspection of hydrogen bonding and hydrophobic interactions with key residues. To further investigate the stability and conformational changes in IL-1β upon binding of selected compounds, MD simulations were performed. Overall, the MD simulations provided insights into the stability and conformational changes of the selected compounds in complex with IL-1β.

Upon examining the chemical structures of the selected hit compounds, it was observed that compound **1**, compound **2**, and compound **3** have flavonoid as their chemical scaffold, whereas compound **4** has a sterol as its chemical scaffold. The existing literature is replete with studies that have demonstrated the anti-inflammatory activity of both flavonoids and sterols [42,43]. However, to best of our knowledge, the compounds identified in this study have not been previously tested against IL-1β. Therefore, it would be intriguing to conduct *in vitro* experiments to assess the potential of the selected hit compounds for mitigating dysregulated expression of IL-1β, an underlying factor contributing to the severity of both COVID-19 and dry eye disease.

## 4. Materials and Methods

### 4.1. Target Preparation

The crystallographic structure of the IL-1β in complex with the IL-1R receptor was retrieved from the Protein DataBank with the accession code of 1ITB [31]. Afterwards, the Protein Preparation Wizard of Molecular Operating Environment (MOE v2019.01) was used to prepare the structure for further analysis [44]. The preparation involved several steps, including assigning the correct bond order, removing unnecessary water molecules, terminal capping, insertion of missing atoms, energy minimization, and assigning partial charges. The AMBER99 force field was used for this preparation. Additionally, missing hydrogen atoms were incorporated into the structure at the typical protonation condition of pH 7. Finally, the prepared structure was saved in PDB format for further analysis.

### 4.2. Database Preparation

A traditional Chinese medicine (TCM) database of ~36,000 chemical compounds was utilized to extract the potential virtual hits against IL-1β. The database is curated with several compounds from various sources, such as herbs, animal products, and minerals used in TCM regimens. The chemical compounds in the database were converted into 3D format (mol2) using the OPENBABEL package [45]. Geometry correction and protonation were performed using MOE, and hydrogens were added and partial charges were applied, as needed. After the initial steps, the compounds underwent energy minimization using the MMFF94x force field to correct the geometry and remove any bad clashes in the compounds. The chemicals were then saved in MOE database format (mdb) for further computation.

### 4.3. Docking-Based Virtual Screening

Through genetic modification, researchers have been able to pinpoint two specific locations (site A and site B) on the surface of the IL-1β protein that are responsible for binding to receptors. It also has potential implications for the development of drugs that can target these binding sites to modulate the activity of IL-1β and potentially treat diseases. Recently, Sardar et al. evaluated the potency of both sites and suggested that site A is a more promising target site for drug design and the discovery of small-molecule inhibitors of IL-1β [32]. Therefore, in this study, we also targeted site A for docking-based virtual screening of the TCM database. Initially, a docking grid was generated by selecting the residues of site A present at the interface of the IL-1β/IL-1R complex, defined as the docking site. The prepared compounds from the TCM database were docked into the defined grid using a protocol previously established by Sardar et al. using MOE software [32]. Briefly, the compounds were docked using the rigid protocol, while Triangular Matcher was used as a placement method. For placement poses, London dG was used as a scoring function, while, for the final refinement of poses, GBVI/WSA dG was used as a rescoring function.

To prioritize the compounds, three different filtration criteria were applied. (i) The compounds were searched for interactions with the crucial residues of site A using the protein–ligand interaction fingerprints (PLIF) module of MOE, resulting in ~5000 compounds. (ii) The second filter was based on the docking score, and the top-ranked compounds with a docking score in the range of −14.9 to −6.0 kcal/mol were selected, resulting in ~700 virtual hits. (iii) The third criteria involved visual analysis of the compounds using the PLIP webserver and Chimera software. This analysis process entailed the visual inspection of hydrogen bonding and hydrophobic interactions with the specific crucial residues and complementarity between the ligand and the binding site of IL-1β. Based on the three criteria used in the multi-step screening process, four compounds were deemed to be the most promising candidates for further investigation and subjected to MD simulation.

### 4.4. Molecular Dynamics Simulation

To investigate the dynamic behavior and stability of the four selected hit compounds within the binding cavity of IL-1β, a molecular dynamics (MD) simulation was carried out by AMBER22 [46]. A total of five systems were prepared, including an IL-1β/IL-1R complex and four ligand-bound protein complexes (compound **1**, compound **2**, compound **3** and compound **4**). The atomic partial charges of the aforementioned compounds were derived via AM1-BCC charge calculation using the Antechamber package. The target protein was parameterized by executing an FF19SB force field, while for the stereochemistry, atom typing and geometric parametrization of the compounds were executed via a GAFF2 force field. Following the preparation, all the systems were solvated by explicit TIP3P water molecules into a periodic box that extended at a distance of 8 Å from the protein atoms. All prepared systems were energy minimized to correct any geometry issues and steric clashes. The minimization was carried out in two steps, with the first 2500 steps using the steepest descent algorithm, followed by the remaining 2500 steps using the conjugate gradient algorithm. Afterwards, the systems were gradually heated from 0 to 300 K within 500 ps, followed by 500 ps of equilibration at 300 K to obtain a stable system. The system was then subjected to NPT equilibration for 1000 ps at 1 atm pressure and 300 K temperature. The final MD production run was performed for 100 ns with an integration time step of 2.0 fs. Trajectories were collected after every 1 ps for subsequent analysis. The CPPTRAJ module within the Amber software was used to analyze the trajectories. Root mean square deviation (RMSD), root mean square fluctuation (RMSF), and radius of gyration (Rg) were calculated for all five simulated systems to predict their dynamic behavior.

## 5. Conclusions

Excessive production of IL-1β has been linked to several inflammatory and autoimmune diseases, including severe COVID-19 and dry eye disease. Therefore, targeting IL-1β signaling pathways is a potential therapeutic strategy for these diseases. In this study, a docking-based screening strategy was employed to identify potential IL-1β inhibitors from the TCM database. The compounds were shortlisted based on their higher binding score than −6.0 kcal/mol, interactions with the crucial residues of site A of IL-1β, and visual inspection of their binding modes. Finally, compounds **1**, **2**, **3**, and **4** with binding scores of −10.10, −11.12, −10.53 and −14.19 kcal/mol, respectively, having flavonoid and sterol as their basic scaffolds, were selected as potential binders of IL-1β. Molecular dynamics simulations were performed to gain insights into the dynamic behavior of protein–ligand interactions. The analysis revealed that four selected compounds were stabilized in an IL-1β pocket with an RMSD up to 4 Å with variable fluctuations and may interfere with the formation of the IL-1β/IL-1R complex. Therefore, these compounds have the potential to be investigated further as potential therapeutic agents for inflammatory and autoimmune diseases. Our study lays the groundwork for further research in this field and the development of potential drugs to combat diseases that involve dysregulated expression of IL-1β as the underlying cause.

## Figures and Tables

**Table 1:** The chemical structure and binding score of selected virtual hit compounds.

Compound Code	Structure	Binding Score (kcal/mol)
Compound **1**	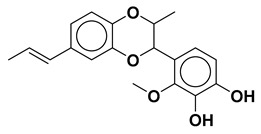	−10.10
Compound **2**	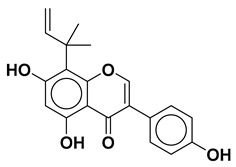	−11.12
Compound **3**	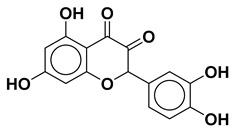	−10.53
Compound **4**	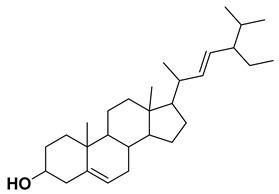	−14.19

## Data Availability

Not applicable.
